# Criteria for the diagnosis of Alzheimer’s disease: Recommendations of the Scientific Department of Cognitive Neurology and Aging of the Brazilian Academy of Neurology

**DOI:** 10.1590/S1980-57642011DN05030002

**Published:** 2011

**Authors:** Norberto Anízio Ferreira Frota, Ricardo Nitrini, Benito Pereira Damasceno, Orestes V. Forlenza, Elza Dias-Tosta, Amauri B. da Silva, Emílio Herrera Junior, Regina Miksian Magaldi, Group Recommendations in Alzheimer’s Disease and Vascular Dementia of the Brazilian Academy of Neurology

**Affiliations:** 1 Medicine Course, University of Fortaleza (Unifor). Neurology Service of Fortaleza General Hospital (HGF), Fortaleza CE, Brazil; 2 Cognitive Neurology and Behavior Group, Hospital das Clínicas, School of Medicine, University of São Paulo (FMUSP). Referral Center for Cognitive Disorders (CEREDIC) of the FMUSP, São Paulo SP, Brazil; 3 Department of Neurology, State University of Campinas (FCM-UNICAMP), Campinas PS, Brazil; 4 Neurosciences Laboratory-LIM 27, Department and Institute of Psychiatry, School of Medicine, FMUSP; 5 Neurologist, Hospital de Base, Federal District, Brasília DF, Brazil; 6 UNINEURO, Recife PE, Brazil; 7 Department of Internal Medicine, School of Medicine of Catanduva, Catanduva SP, Brazil; 8 Geriatrics Service, Hospital das Clínicas, FMUSP and Referral Center for Cognitive Disorders, Hospital das Clínicas, FMUSP, São Paulo SP, Brazil

**Keywords:** dementia, Alzheimer’s disease, mild cognitive impairment, diagnosis, consensus guidelines, Brazil

## Abstract

This consensus prepared by the Scientific Department of Cognitive Neurology and Aging of the Brazilian Academy of Neurology is aimed at recommending new criteria for the diagnosis of dementia and Alzheimer’s disease (AD) in Brazil. A revision was performed of the proposals of clinical and of research criteria suggested by other institutions and international consensuses. The new proposal for the diagnosis of dementia does not necessarily require memory impairment if the cognitive or behavioral compromise affects at least two of the following domains: memory, executive function, speech, visual-spatial ability and change in personality. For the purpose of diagnosis, AD is divided into three phases: dementia, mild cognitive impairment and pre-clinical phase, where the latter only applies to clinical research. In the dementia picture, other initial forms were accepted which do not involve amnesia and require a neuroimaging examination. Cerebrospinal fluid biomarkers are recommended for study, but can be utilized as optional instruments, when deemed appropriate by the clinician.

## Abstract (pt)

Este consenso realizado pelo Departamento Científico de Neurologia Cognitiva e do Envelhecimento da Academia Brasileira de Neurologia tem como objetivo recomendar novos critérios para diagnóstico de demência e doença de Alzheimer (DA) no Brasil. Foi realizada revisão das propostas de critérios clínicos e de pesquisa sugeridas por outras instituições e consensos internacionais. A nova proposta para o diagnóstico de demência exige o comprometimento funcional e cognitivo, atingindo este último pelo dois dos seguintes cinco domínios a seguir: memória, função executiva, linguagem, habilidade visual-espacial e alteração de personalidade. No diagnóstico de DA, dividiu-se a mesma em três fases: demência, comprometimento cognitivo leve e pré-clínica, sendo esta última somente para pesquisa clínica. No quadro de demência, foram aceitas outras formas de início que não a amnéstica e incluída a necessidade de exame de neuroimagem. O diagnóstico do comprometimento cognitivo leve é clínico, podendo, em situações de pesquisas, serem utilizados marcadores biológicos buscando maior probabilidade de evolução para DA.

## Introduction

In 2005, the Department of Cognitive Neurology and Aging of the Brazilian Academy of Neurology^1^ met to formulate the first recommendations for the diagnosis of Alzheimer’s disease (AD) in Brazil. On this occasion, the criteria of the DSM IV^2^ were recommended for the diagnosis of dementia and those of the NINCDS-ADRDA^3^ for the diagnosis of AD given they were the most commonly used and had the highest sensitivity and specificity. In recent years however, there have been important advances in the understanding of AD, such as the observation of various clinical spectra besides amnesia, and improved *in vivo* detection of the physiopathological processes involved in the disease, making it necessary to review these criteria.^4^

Neuropathological studies have demonstrated that pathological alterations found in AD can be present in asymptomatic individuals.^5^ The use of biomarkers in recent years has shown that the physiopathological process of AD can be identified in asymptomatic individuals as well as in patients with established dementia.^4,6^

Currently available biomarkers for AD make it possible to detect the peptide amyloid β (Aβ-42) and tau protein, which show correlation with the pathology of AD.^6^ Alteration in the peptide Aβ-42, albeit a decrease in its concentration in spinal fluid or the identification of deposits of the peptide in cerebral tissues can be detected by new molecular neuroimaging methods of positron emission tomography (PET). Although possibly occurring in other diseases, these peptide changes are more specific and appear earlier (up to 10 years before the emergence of first symptoms) than elevations in the tau protein or phosphorylated tau. Alterations in tau protein, as well as hippocampal atrophy visualized on magnetic resonance (MR) imaging, and hypometabolism of glucose detected by the FDG-PET method, appear to be related to neuronal injury/damage. Alterations in neuronal damage markers occur several years before the emergence of clinical symptoms.^4^ The occurrence of alterations in both amyloid and neuronal damage markers shows a good correlation with AD and increases the probability of reaching a definitive diagnosis. However, the routine use of amyloid markers is not indicated due to the lack of standardization among laboratories, poorly-defined cut-off points as well as poor availability of the tests, since their use is restricted to research settings.^4^

In previous criteria, AD was only diagnosed in the presence of dementia, while in the new proposals AD can be diagnosed in three phases or stages, namely: pre-clinical AD, mild cognitive impairment (MCI) due to AD, and dementia, where the diagnosis of the pre-clinical phase should be restricted to research settings.

In 2007, Dubois et al. proposed criteria for the clinical diagnosis of AD for the purpose of research, utilizing supplementary diagnostic methods: MR, PET or spinal fluid biomarkers (Aβ-42 and tau), aimed at achieving greater specificity and earlier diagnosis.^7^ These authors suggested a new definition for the disease, not restricted only to the dementia phase, but allowing for its detection in pre-clinical stages based on the presence of alterations on MR, PET and biomarker evaluations indicating potential physiopathological changes of AD in asymptomatic patients.^8^

During meetings in 2009, the Working Group of the *National Institute on Aging* (NIA) *and Alzheimer’s Association* (AA) prepared new recommendations for the clinical diagnosis of AD which were presented at the 2010 *International Conference on Alzheimer’s Disease*. The recommendations were available for appraisal in the summer of 2010, revised and subsequently published.^4,9-11^

The recommendations for the diagnosis of AD in Brazil that follow, were formulated by the members of the Department of Cognitive Neurology and Aging of the Brazilian Academy of Neurology based on the advances made in recent years described above, but have undergone some modifications and adaptations that are presented below and emphasized in the conclusions.

## Diagnosis of dementia

The DSM-IV^2^ criteria for the diagnosis of dementia require memory impairment. However, various diseases involve cognitive decline and functional loss, such as frontotemporal dementia, vascular dementia and dementia with Lewy bodies, and may not show compromise of memory in initial phases.^12,13^ Thus, these criteria now need revising to accommodate these forms of dementia.

Proposals for utilizing compromise in two or more cognitive domains, independent of memory, have been suggested by other authors.^14,15^ At the meeting of the Working Group of the NIA and AA, proposals were put forth for new criteria for dementia which, due to the non-requirement of memory impairment, make them applicable to other etiologies. These criteria are recommended for application in Brazil by the Brazilian Academy of Neurology.

### I. MAIN CLINICAL CRITERIA FOR THE DIAGNOSIS OF DEMENTIA (OF ANY ETIOLOGY)

#### 1. Dementia diagnosis is designated when there are cognitive or behavioral (neuropsychiatric) symptoms that:

1A. Interfere with the ability to work or to carry out usual activities.
1B. Represent decline in relation to pre-morbid levels of functioning and performance.
1C. Cannot be explained by *delirium* (acute confusional state) or major psychiatric disease,

#### 2. Cognitive compromise is detected and diagnosed based on a combination of:

2A. Anamnesis with patient and close family members/friends who have knowledge of the patient’s history; and
2B. Objective cognitive evaluation through a brief cognitive examination of mental state or a neuropsychological assessment. Neuropsychological assessment should be done when anamnesis and the brief cognitive examination carried out by a clinician are insufficient to reach a reliable diagnosis.

#### 3. Cognitive or behavioral compromise affects at least two of the following domains:

3A. Memory, characterized by compromise of the capacity to acquire or recall recent information, with symptoms that include: repetition of the same questions or subjects, forgetting of events, agreements or place where belongings are kept.
3B. Executive functions, characterized by compromise in reasoning, carrying out complex tasks and judgment, with symptoms such as: poor comprehension of risk situations and reduced ability to take care of finances, make decisions and plan complex or sequential activities.
3C. Visual-spatial abilities, with symptoms that include: inability to recognize faces or common objects and find objects in the visual field, difficulty handling utensils and dressing oneself for reasons other than visual or motor deficiency.
3D. Speech (expression, comprehension, reading and writing), with symptoms that include: difficulty in finding and/or understanding words, errors in speaking and writing, and exchange of words or phonemes, not explicable by a sensory or motor deficit.
3E. Personality or behavior, with symptoms that include changes in mood (instability, uncharacteristic fluctuations), agitation, apathy, disinterest, social isolation, loss of empathy, disinhibition, and obsessive, compulsive or socially unacceptable behavior.

### II. DEMENTIA OF ALZHEIMER’S DISEASE: CENTRAL CLINICAL CRITERIA

#### 1. Dementia of probable Alzheimer’s disease (modified from McKhann et al., 2011)

Meets criteria for dementia and has the following additional characteristics:

1. Insidious onset (months or years).
2. Clear history or observation of cognitive decline.
3. Initial and more prominent cognitive deficits in one of the following categories:
  3A. Amnesic presentation (should have another affected domain).
  3B. Non-amnesic presentation (should have another affected domain):
    – Speech (remembering words).
    – Visual-spatial (spatial cognition, agnosia of objects or faces, simultaneous agnosia and alexia).
    – Executive functions (alteration in reasoning, judgment and resolution of problems).
4. Tomography or preferentially, magnetic resonance, of the head should be done to exclude other diagnostic possibilities or comorbidities, particularly cerebral vascular disease.
5. The diagnosis of dementia of probable AD should not be applied when there is:
  5A. Evidence of significant cerebrovascular disease defined by history of cerebral vascular accident (CVA) temporally related to the onset or worsening of cognitive compromise, or presence of multiple or extensive infarcts, or marked lesions in white matter evidenced by neuroimaging examinations; or
  5B. Central characteristics of dementia with Lewy bodies (visual hallucinations, parkinsonism and cognitive fluctuation); or
  5C. Prominent characteristics of the behavioral variant of frontotemporal dementia (hyperorality, hypersexuality, perseverance); or
  5D. Prominent characteristics of primary progressive aphasia manifesting as the semantic variant (also called semantic dementia, with fluent discourse, anomia and difficulties with semantic memory) or as the non-fluent variant, with substantial agrammatism; or
  5E. Evidence of another concomitant and active disease, neurological or non-neurological, or of the use of medication that can have a substantial effect on cognition.
    The following items, when present, increase the degree of reliability of the clinical diagnosis of dementia of probable AD:
    - Evidence of progressive cognitive decline, found on successive assessments.
    - Proof of the presence of causative genetic mutation (genes of APP and presenilins 1 and 2).
    - Positivity of biomarkers that reflect the pathogenic process of AD (molecular markers by PET or spinal fluid, or structural and functional neuroimaging).

The occurrence of item (a) confirms the existence of a degenerative mechanism, despite not being specific for AD.

#### 2. Dementia of possible Alzheimer’s disease

The diagnosis of dementia of possible AD should be designated when the patient meets the clinical diagnostic criteria for dementia of AD by presenting some of the signs and symptoms below:

1. Atypical course: abrupt onset and/or pattern of evolution distinct from that usually observed i.e. slowly progressive.
2. Mixed presentation: there is evidence of other etiologies as described in item 5 of the criteria of dementia of probable AD (concomitant cerebrovascular disease, characteristics of dementia with Lewy bodies, other neurological disease or a non-neurological comorbidity or use of medication that can have a substantial effect on cognition)
3. Insufficient details of history of the establishment and development of the disease.

#### 3. Dementia of definite Alzheimer’s disease

Meets the clinical and cognitive criteria for dementia of AD. Neuropathological examination demonstrates the presence of AD pathology according to the criteria of the NIA and *Reagan Institute Working* Group.^16^

### III. DIAGNOSIS OF MILD COGNITIVE IMPAIRMENT (MCI) DUE TO AD (MODIFIED FROM ALBERT ET AL., 2011)

There are two combinations of criteria that can be utilized for the diagnosis of MCI due to AD.

1. Central clinical criteria: for use in clinical practice, without the need for tests or highly specialized procedures.
2. Clinical research criteria: which incorporate information obtained from the use of biomarkers and are specifically intended for research purposes, specialized centers and clinical trials.

#### 1. Central clinical criteria

##### 1.1. CLINICAL AND COGNITIVE CHARACTERISTICS

- Complaint of cognitive alteration reported by the patient, close relative/friend or health care professional.
- Evidence of compromise in one or more cognitive domains typically including memory, obtained through evaluation covering the following cognitive domains: memory, executive function, speech and visual-spatial abilities, or neuropsychological examination.
- Preservation of independence in daily life activities. Can have slight problems in performing complex previously habitual tasks, such as paying bills, preparing a meal or shopping. The patient can take longer, be less efficient and make more mistakes in carrying out these activities. However, the patient is still able to maintain independence with minimal assistance.
- Does not meet the criteria for dementia.

There is still no consensus on which batteries of tests should be utilized for the diagnosis of cognitive compromise in MCI. Neuropsychological tests should preferably be used because they are more sensitive. There is no norm for cut-off values, but suggestions have been made of between 1 and 1.5 standard deviations below expected levels. Cognitive screening tests, such as the capacity to write down and recall an address, or remember objects shown at office visits and then hidden, can be used in clinical practice, despite being less sensitive.^9^

##### 1.2. ETIOLOGY CONSISTENT WITH AD

- Discard other systemic or neurological diseases that could be responsible for cognitive decline.
- Evidence of longitudinal decline of cognition consistent with natural development of AD, when possible.
- History consistent with family AD.

Other neurological diseases that can lead to cognitive decline (trauma, vascular, medications) should be ruled out. Parkinsonian symptoms, important cardiovascular risk factors and significant vascular alterations on neuroimaging examinations, besides prominent signs of frontotemporal lobe degeneration should be considered, as suggested in the diagnosis of dementia of probable AD.^9^

The presence of dominant autosomal genetic alterations of AD in family members of the patient make it more likely that MCI is the cause of the disease.

#### 2. Criteria of clinical research for MCI due to AD

Once meeting the clinical criteria of MCI due to AD, the information obtained by biomarkers can confer different degrees of probability of the etiology of AD. This classification of probability needs to be tested in future studies before being used in clinical practice.^9^

- **High probability**

– Biomarkers of Aβ and neuronal lesion/damage are positive.

- **Intermediate probability**

– Only one of the modalities is positive and the other was not tested.

- **Low probability**

– Biomarkers of Aβ and of neuronal lesion/damage are negative.

- **Inconclusive data:**

– Uncharacteristic or conflicting results (Aβ biomarker positive and that of neuronal lesion/damage negative or vice-versa).

The degree of certainty of high probability is also related to the greater incidence and shorter development time for dementia. The absence of both types of biomarkers leads to consideration of other etiologies (non-AD) for the picture of MCI.

### IV. DIAGNOSIS OF PRE-CLINICAL ALZHEIMER’S DISEASE FOR CLINICAL RESEARCH

For the purpose of clinical research, it is possible to propose the diagnosis of AD before the appearance of clinical symptoms based on information obtained through the use of biomarkers, as proposed by Sperling and coworkers (2011). However, this proposal still requires experimental validation through longitudinal studies.

- **Stage 1: Asymptomatic cerebral amyloidosis**

– Elevated capture of βA marker on PET.
– Reduction of βA-42 in spinal fluid.

- **Stage 2: Amyloidosis + initial neurodegeneration**

– Markers of β-amyloid deposition positive.
– Neuronal dysfunction on FDG-PET/fMRI.
– Increased tau/phosphor tau in spinal fluid.
– Reduction of cortical thickness/hippocampal atrophy by MR.

- **Stage 3: Positivity for amyloid + evidence of neurodegeneration + subtle cognitive decline (high cognitive demand tests)**

– Meeting requirements of stages 1 and 2.
– Evidence of previous subtle alteration in cognitive level.
– Low performance on more complex cognitive tests.
– Not meeting the criteria for MCI.

## Revealing the diagnosis

The question of disclosing the diagnosis warrants inclusion among the recommendations. In recent decades, there has been a major shift in diagnosis disclosure from a paternalistic stance to one of greater autonomy of patients. Some medical institutions always advise revealing the diagnosis of demential pictures to patients whenever possible, but cultural, individual and regional factors should be taken into account.^17^

The percentage of family members of patients with AD who would like to have the diagnosis revealed to the patient ranges from 17 to 76% depending on the country of study.^17^ In Brazil, 58% of family members of patients were found to be in favor of revealing the diagnosis,^18^ which is routinely done by 44.7% of doctors.^19^ Family members and physicians not wishing to reveal the diagnosis more frequently would like such a diagnosis revealed to themselves if they were the patient (90% and 76.8%, respectively).^18,19^ Family members with a higher level of education^18^ and doctors with longer periods of training^19^ appear to be more in favor of not revealing the diagnosis.

The main reason given for not wanting the diagnosis revealed is the negative impact on the patient. Nonetheless, there is still much to investigate on this subject, in as far as the impact of revealing the diagnosis has not been sufficiently studied. The opinions of patients, their family and doctors on the best practice tends to vary over time, perhaps pointing to the need for periodic reevaluation of the approach in a dynamic process which should be modified on account of the impact of new treatments. Individualizing the approach on this issue appears to be the best strategy given the current state of understanding.^20^

## Conclusions

These new recommendations for the diagnosis of AD represent an advance in relation to those of 2005. Firstly, the condition designated as AD based on the criteria of 2005 is now called dementia of AD, while the general designation now encompasses the pre-clinical phase and MCI due to AD.

For the diagnosis of dementia, there is no longer the compulsory need of memory impairment, still required by DSM IV,^2^ DSM-IIIR^21^ and CID -10^22^ and recommended in 2005. This modification is very important since it allows the classification of cases of frontotemporal dementia, vascular dementia and other forms of dementia that have already been included under the designation of dementia, although without consensus on recommendations and criteria followed.

Unlike previous criteria, the diagnosis of dementia or AD only needs confirmation by means of neuropsychological assessment in cases when anamnesis and cognitive evaluation done by a clinician are insufficient for diagnosis. The limitation in age of onset of between 40 and 90 years has also been dropped from the current criteria.

The main difference between our recommendations and the proposals by NIA and AA for the diagnosis of dementia of AD was the inclusion of our recommendations of the need for imaging examinations, tomography of the head or preferentially magnetic resonance of the head, to exclude other etiologies or comorbidities. In fact, we believe that this necessity is implicit in the exclusion criteria adopted by the NIA and AA, which we also followed.

The inclusion of biomarkers in the diagnosis was recommended but only in clinical research settings. These new methods are discussed in detail in the section on supplementary examinations. There is a need for further studies to validate the criteria of MCI associated with biomarkers, as well as the criteria of the pre-symptomatic phase of AD. However, optional instruments can be employed when considered appropriate by the clinician.

## GROUP RECOMMENDATIONS IN ALZHEIMER’S DISEASE AND VASCULAR DEMENTIA OF THE BRAZILIAN ACADEMY OF NEUROLOGY

**Ana Cláudia Ferraz** [Serviço de Neurologia do Hospital Santa Marcelina (SP)]; **Analuiza Camozzato de Pádua** [Universidade Federal de Ciências da Saúde de Porto Alegre (UFCSPA); Hospital de Clínicas de Porto Alegre (UFRGS) (RS)]; **Antonio Lúcio Teixeira** [Departamento de Clínica Médica, Faculdade de Medicina da Universidade Federal de Minas Gerais, Belo Horizonte (MG)]; **Ayrton Roberto Massaro** [Instituto de Reabilitação Lucy Montoro (SP)]; **Carla Tocquer** [Universidade Federal do Rio de Janeiro (RJ)]; **Carlos Alberto Buchpiguel** [Departamento de Radiologia, Faculdade de Medicina da Universidade de São Paulo (SP)]; **Cássio Machado C. Bottino** [Programa Terceira Idade, Instituto de Psiquiatria do Hospital das Clínicas da Faculdade de Medicina da Universidade de São Paulo (FMUSP) (SP)]; **Charles André** [Faculdade de Medicina - UFRJ; SINAPSE Reabilitação e Neurofisiologia (RJ)]; **Cláudia C. Godinho** [Serviço de Neurologia do Hospital de Clínicas de Porto Alegre, Universidade Federal do Rio Grande do Sul (RS)]; **Cláudia Sellitto Porto** [Grupo de Neurologia Cognitiva e do Comportamento da Faculdade de Medicina da USP (SP)]; **Delson José da Silva** [Núcleo de Neurociências do Hospital das Clínicas da Universidade Federal de Goiás (UFG); Instituto Integrado de Neurociências (IINEURO), Goiânia (GO)]; **Denise Madeira Moreira** [Departamento de Radiologia Faculdade de Medicina - UFRJ; Setor de Radiologia - INDC - UFRJ (RJ)]; **Eliasz Engelhardt** [Setor de Neurologia Cognitiva e do Comportamento - INDC - CDA/IPUB - UFRJ (RJ)]; **Francisco de Assis Carvalho do Vale** [Universidade Federal de São Carlos (UFSCar), Departamento de Medicina (DMed) (SP)]; **Gabriel R. de Freitas** [Instituto D’or de Pesquisa e Ensino; Universidade Federal Fluminense (RJ)]; **Hae Won Lee** [Instituto de Radiologia, Hospital das Clínicas da Faculdade de Medicina da Universidade de São Paulo e Hospital Sírio-Libanês (SP)]; **Ivan Hideyo Okamoto** [Departamento de Neurologia e Neurocirurgia; Instituto da Memória - Universidade Federal de São Paulo - UNIFESP (SP)]; **Jerusa Smid** [Grupo de Neurologia Cognitiva e do Comportamento do Hospital das Clínicas da Faculdade de Medicina da Universidade de São Paulo (FMUSP) (SP)]; **João Carlos Barbosa Machado** [Aurus IEPE - Instituto de Ensino e Pesquisa do Envelhecimento de Belo Horizonte; Faculdade de Ciências Médicas de Minas Gerais (FCMMG), Serviço de Medicina Geriátrica do Hospital Mater Dei (MG)]; **José Antonio Livramento** [Laboratório de Investigação Médica (LIM) 15, Faculdade de Medicina da Universidade de São Paulo (SP)]; **José Luiz de Sá Cavalcanti** [Departamento de Neurologia - INDC - UFRJ; Setor de Neurologia Cognitiva e do Comportamento - INDC - UFRJ (RJ)]; **Letícia Lessa Mansur** [Grupo de Neurologia Cognitiva e do Comportamento do Departamento de Neurologia da FMUSP; Departamento de Fisioterapia, Fonoaudiologia e Terapia Ocupacional da Faculdade de Medicina da USP (SP)]; **Liana Lisboa Fernandez** [Departamento de Ciências Básicas da Saúde, Fundação Universidade Federal de Ciências da Saúde de Porto Alegre (RS)]; **Márcia Lorena Fagundes Chaves** [Serviço de Neurologia do Hospital de Clínicas de Porto Alegre, Universidade Federal do Rio Grande do Sul (RS)]; **Márcia Radanovic** [Laboratório de Neurociências - LIM27, Departamento e Instituto de Psiquiatria da Faculdade de Medicina da Universidade de São Paulo (FMUSP) (SP)]; **Márcio Luiz Figueredo Balthazar** [Universidade Estadual de Campinas (UNICAMP), Faculdade de Ciências Médicas (FCM), Departamento de Neurologia (SP)]; **Maria Teresa Carthery-Goulart** [Grupo de Neurologia Cognitiva e do Comportamento do Departamento de Neurologia da Faculdade de Medicina da USP; Centro de Matemática, Computação e Cognição, Universidade Federal do ABC (SP)]; **Mônica S. Yassuda** [Grupo de Neurologia Cognitiva e do Comportamento do Departamento de Neurologia da Faculdade de Medicina da USP; Departamento de Gerontologia, Escola de Artes, Ciências e Humanidades da USP (EACH/USP Leste) (SP)]; **Nasser Allam** [Universidade de Brasília (UnB), Laboratório de Neurociências e Comportamento, Brasília (DF)]; **Paulo Caramelli** [Departamento de Clínica Médica, Faculdade de Medicina da Universidade Federal de Minas Gerais, Belo Horizonte (MG)]; **Paulo Henrique Ferreira Bertolucci** [Universidade Federal de São Paulo (UNIFESP), Setor de Neurologia do Comportamento - Escola Paulista de Medicina, São Paulo (SP)]; **Renata Areza-Fegyveres** [Grupo de Neurologia Cognitiva e do Comportamento do Hospital das Clínicas da Faculdade de Medicina da Universidade de São Paulo (FMUSP) (SP)]; **Renato Anghinah** [Grupo de Neurologia Cognitiva e do Comportamento do Hospital das Clínicas da Faculdade de Medicina da Universidade de São Paulo (FMUSP); Centro de Referência em Distúrbios Cognitivos (CEREDIC) da FMUSP (SP)]; **Rodrigo Rizek Schultz** [Setor de Neurologia do Comportamento do Departamento de Neurologia e Neurocirurgia da Universidade Federal de São Paulo, Núcleo de Envelhecimento Cerebral (NUDEC) - Instituto da Memória (UNIFESP) (SP)]; **Rogério Beato** [Grupo de Pesquisa em Neurologia Cognitiva e do Comportamento, Departamento de Medicina Interna, Faculdade de Medicina, UFMG (MG)]; **Sonia Maria Dozzi Brucki** [Grupo de Neurologia Cognitiva e do Comportamento da Faculdade de Medicina da Universidade de São Paulo; Centro de Referência em Distúrbios Cognitivos (CEREDIC) da FMUSP; Hospital Santa Marcelina (SP)]; **Tânia Novaretti** [Faculdade de Filosofia e Ciências, Campus de Marília, da Universidade Estadual Paulista (UNESP) (SP)]; **Valéria Santoro Bahia** [Grupo de Neurologia Cognitiva e do Comportamento do Hospital das Clínicas da Faculdade de Medicina da Universidade de São Paulo (FMUSP) (SP)]; **Ylmar Corrêa Neto** [Universidade Federal de Santa Catarina (UFSC), Departamento de Clínica Médica, Florianópolis (SC)].
